# Betel Chewing and Arecoline Affects Eotaxin-1, Asthma and Lung Function

**DOI:** 10.1371/journal.pone.0091889

**Published:** 2014-03-21

**Authors:** Tsu-Nai Wang, Ming-Shyan Huang, Meng-Chih Lin, Tsai-Hui Duh, Chih-Hung Lee, Chin-Chou Wang, Ping-Ho Chen, Shang-Lun Chiang, Chau-Chyun Sheu, Vincent Chin-Hung Chen, Chao-Chien Wu, Cleusa P. Ferri, Robert Stewart, Ying-Chin Ko

**Affiliations:** 1 Department of Public Health, College of Health Science, Kaohsiung Medical University, Kaohsiung, Taiwan; 2 Center of Excellence for Environmental Medicine, Kaohsiung Medical University, Kaohsiung, Taiwan; 3 Division of Pulmonary and Critical Care Medicine and Geriatric Medicine, Department of Internal Medicine, Kaohsiung Medical University Hospital, College of Medicine, Kaohsiung Medical University, Kaohsiung, Taiwan; 4 Division of Pulmonary and Critical Care Medicine, Department of Medicine, Chang Gung Memorial Hospital-Kaohsiung Medical Center, Chang Gung University College of Medicine, Kaohsiung, Taiwan; 5 Department of Medicinal and Applied Chemistry, Kaohsiung Medical University, Kaohsiung, Taiwan; 6 Department of Dermatology, Kaohsiung Chang Gung Memorial Hospital and Chang Gung University College of Medicine, Kaohsiung, Taiwan; 7 School of Dentistry, College of Dental Medicine, Kaohsiung Medical University, Kaohsiung, Taiwan; 8 Environment-Omics-Diseases Research Center, China Medical University Hospital, Taichung, Taiwan; 9 Graduate Institute of Clinical Medical Science, China Medical University, Taichung, Taiwan; 10 Division of Pulmonary and Critical Care Medicine, Department of Internal Medicine, Kaohsiung Medical University Hospital, Faculty of Medicine, College of Medicine, Kaohsiung Medical University, Kaohsiung, Taiwan; 11 Department of Psychiatry, School of Medicine, Institute of Medicine, Chung Shan Medical University, The Department of Psychiatry, Chung Shan Medical University Hospital, Taichung. Taiwan; 12 Universidade Federal de São Paulo – Psychobiology Department, São Paulo, Brazil; 13 King's College London, Institute of Psychiatry, London, United Kingdom; University ofTennessee Health Science Center, United States of America

## Abstract

**Background:**

Betel nut is commonly used in many countries. Despite evidence suggesting an association with asthma, few studies have investigated the connection between betel nut use and asthma; thus, the underlying mechanism for the association with asthma is also unclear. The aim of this study was to investigate the association between betel chewing and asthma as well as the associations of plasma arecoline (a biomarker for exposure) and eotaxin-1 (a potential mediator) with asthma and lung function.

**Methods:**

We recruited 600 hospital-based asthmatic patients and 1200 age- and gender-matched community controls in southern Taiwan. To clarify the mechanism of action for eotaxin-1 in the association between betel chewing and asthma, we also designed an *in vitro* experiment to study the functional associations between arecoline exposure and eotaxin-1 levels.

**Results:**

A significant association was found between asthma and current betel chewing (adjusted odds ratio 2.05, 95% CI = 1.12–3.76), which was independent of potential confounders but was attenuated following adjustment for eotaxin-1. Arecoline and eotaxin-1 levels were positively correlated (Spearman r = 0.303, p = 0.02), while arecoline and arecaidine were negatively correlated with lung function. Functionally, arecoline alone does not induce eotaxin-1 release *in vitro* from dermal and gingival fibroblasts. However, in the presence of IL-4 and TNF-alpha, arecoline at 100 μg/ml induced more eotaxin-1 release than arecoline at 0 μg/ml (2700±98 pg/ml vs 1850±142 pg/ml, p = 0.01 in dermal fibroblast cells, and 1489±78 pg/ml vs 1044±95 pg/ml, p = 0.03 in gingival fibroblast cells, respectively).

**Conclusion:**

Betel chewing is associated with asthma in this population, with arecoline induction of eotaxin-1 supported as a plausible causal pathway.

## Introduction

Betel nut is a common substance used by an estimated 600 million people worldwide [1], particularly in India, the South Pacific Islands and Southeast Asia. Betel nut is also widely used in Asian migrant populations in the USA [2], UK and Europe [3]. Arecoline, a cholinergic alkaloid, constitutes 0.15–0.67% of the dry weight of betel quid and has been found to be associated with a variety of outcomes, including esophageal cancer, cardiovascular disease, diabetes, and asthma [4]-[6]. Betel quid chewing is also well-recognized as an independent risk factor for oral cancers [7], [3]. The main alkaloids contained in areca nut are arecoline and arecaidine, which have been supported as exposure biomarkers for a the habit of betel chewing [8].

Considering the widespread exposure and potential risk, few studies have investigated the relationship between betel chewing and asthma. Kiyingi found that 11 of 20 asthmatic patients who had ceased betel chewing reported stopping because of aggravation of asthma symptoms. Of 34 patients who still chewed, 22 also reported asthma attacks precipitated by betel chewing [9]. Taylor et al. reported that two patients hospitalized with asthma had been chewing betel nut immediately before the attacks, and they also performed a double-blind challenge test of arecoline inhalation, which was found to cause bronchoconstriction in six of seven people with asthma and one of six healthy controls [10]. However, not all studies have found this reaction [11].

Asthma is a chronic inflammatory disease that is mediated by a number of chemokines. Eotaxin-1 belongs to the CC chemokine family that attracts circulating Th2-lymphocytes and eosinophils from the blood stream to the inflammatory foci. The expression of eotaxin-1 protein and mRNA is significantly increased in the epithelium of airway, proportional to eosinophil infiltration in asthmatic patients [12]. One animal study has reported that smoke increases the levels of eotaxin-1 expression under co-exposure to other allergens, leading to airway inflammation [13]. In the present study, we suggested that the mechanisms of exposure to smoke and arecoline may implicate similar mechanisms of asthma pathogenesis.

Therefore, we investigated the association between betel chewing and asthma and investigated the role of arecoline and eotaxin-1 in this association. We hypothesized that eotaxin-1 would play a mediating role in the association between betel chewing and asthma and carried out a supplementary *in vitro* experiment to study the functional associations between arecoline exposure and eotaxin-1 levels. To our knowledge, this is the first study to investigate the association between betel chewing, eotaxin-1 concentrations and asthma and lung function.

## Materials and Methods

### Ethics Statement

This study was approved by the Institutional Review Board (IRB) and performed in two medical centers in southern Taiwan: Kaohsiung Medical University Hospital (KMUH) and Chang-Gung Memorial Hospital (CGMH). These two hospitals provide comprehensive medical services to patients of diverse socioeconomic status in their geographic area. Informed written consent was obtained from all subjects.

### Case participants with asthma

We designed a case-control study and an *in vitro* experiment to study the association between betel chewing, eotaxin-1 and asthma and lung function. An enrollment system for cases was established in the Division of Pulmonary and Critical Care Medicine of CGMH and KMUH, so that patients with asthma could be identified and offered to participate in a previous study [14]. In our study, all of the patients received a pulmonary function test, including bronchodilator testing. Patients were diagnosed as having asthma if they had the following symptoms, episodic breathlessness, cough, wheezing and chest tightness, according to the Global Initiative for Asthma (GINA) guidelines, and/or they demonstrated that at least a 12% and ≥200 ml increase in FEV1 from the pre-bronchodilator value by spirometry testing [15], [16]. In this study, 693 adults with asthma who were more than 20 years old were approached as outpatients of the Division of Pulmonary and Critical Care Medicine at two medical centers. Of these 693 adults, 16 refused to participate (response rate 97.7%) and a further 77 were excluded because they had emphysema, chronic airway obstruction, tuberculosis, pneumonia or cancer (n = 62), or incomplete data regarding their history of betel consumption (n = 15). Therefore, a case sample of 600 (255 males and 345 females) was analyzed.

### Control participants

In selecting the community control population, the basis for selection was residence in the same geographic areas as the cases. In the context of free health examinations, the community controls were recruited at local health stations by advertising in five communities. An expected sample size of 1500 people aged more than 20 years old was approached, of whom 1428 agreed to participate (534 male, 894 female, response rate 95.2%). Of these, a further 98 were excluded because of a previous history of physician-diagnosed asthma, emphysema, chronic airway obstruction, tuberculosis, pneumonia or cancer (n = 78) or incomplete data regarding betel consumption (n = 20). From a list of all potential controls, the control participants were then matched by age (±5 year) and gender to cases in a ratio of 2 controls to 1 case. Therefore, 1200 controls (510 males and 690 females) were selected from the 1330 eligible participants on the basis of the most recent recruitment.

The participants completed a questionnaire and underwent lung function tests, the measurements of high-sensitivity C-Reactive Protein (hs-CRP) and eotaxin-1, and evaluations of betel chewing; some descriptions of these measurements are provided in File S1.

### Analysis of arecoline and arecaidine levels

A low prevalence of betel chewing was anticipated in Taiwanese females, and indeed, only five females reported current betel chewing in the present study. Therefore, we only selected male current betel chewers (23 men with asthma and 35 male controls) to investigate the arecoline and arecaidine levels. Of these, 2 cases and 3 controls had insufficient plasma for arecoline and arecaidine assays. Therefore, 21 males with asthma and 32 male controls with current betel chewing were compared with respect to these levels. We further selected 42 male asthma cases and 52 male controls with no betel chewing by a 1∶1-2 matching to case and control betel users by age. Plasma specimens were stored at −70°C until they were needed for use in the assays. A liquid chromatography–tandem mass spectrometry (LC–MS–MS) analysis was used to detect plasma arecoline and arecaidine as previously published [8].

### Dermal and gingival fibroblast cultures

We obtained human normal dermal or gingival fibroblasts from normal human skin specimens from routine skin and oral cavity surgery, respectively. Dermal or gingival tissues were obtained and cut to 1–2 mm^3^ and then incubated in a culture dish with Dulbecco's Modified Eagle's Medium (DMEM) containing 10% Fetal bovine serum (FBS) for 7 days to harvest fibroblasts. The third passage of human fibroblasts was seeded. We harvested the dermal fibroblasts and stimulated them with IL-4 and TNF-alpha, both of which were standard agents to induce the robust release of eotaxin-1 from fibroblasts [17], [18]. Fibroblasts were treated with IL-4 (50 ng/ml) and TNF-alpha (100 ng/ml) (both from Peprotech, Rock Hill, NJ) for 72 hours to induce eotaxin-1 release. Cells were also pretreated with different concentrations of arecoline in the first 24 hours where indicated. Each treatment condition was assessed in triplicate in cells.

### Statistical Analysis

The two groups were compared using χ^2^ and t-tests to assess the statistical significance. Because of the skewed distributions of IgE, hs-CRP and eotaxin-1 levels, logarithmical transformations were performed. To control for potential confounding effects, adjusted odds ratios with 95% confidence intervals were calculated for each risk factor using logistic regression. The Sobel-Goodman test was used to test the mediation effect. One-way ANOVA and least significant difference (LSD) multiple comparison tests were used to compare arecoline and eotaxin-1 levels between the four groups of male cases and controls with and without betel chewing, and eotaxin-1 release was compared between cells treated with different doses of arecoline. Spearman correlation coefficients (R-values) were calculated to assess the associations between arecoline, arecaidine, and lung function. The population attributable fraction was estimated by the following formula: frequency of betel chewing in the case group × [(odds ratio–1)/odds ratio], where the odds ratio for the effect of betel chewing on asthma was estimated based on the multiple logistic regression model. We performed statistical analyses using the software package SPSS 14.0.

## Results

In unadjusted analyses (Table 1), the 600 case participants had higher IgE levels compared to the control group. Alcohol consumption behavior was not different between the two groups, but smoking and betel chewing were more common in the case group. In further unadjusted analyses (Table 2), the association with current consumption was confirmed in both men and women, and asthma was also associated with longer durations of betel use, although not with the age of first use or use, as defined by ‘pack x years’. Only three cases reported an age of first betel chewing that was later than their reported age of asthma onset, and the mean±SD period between first betel chewing and asthma diagnosis was 18.6±14.5 years.

**Table 1:** Characteristics of the case and control participants.

	Asthma cases (n = 600)	Community controls (n = 1200)	p-value
Male/female	255/345	510/690	1.00
Mean (SD) age	52.49±14.55	51.98±13.08	0.47
Mean (SD) IgE (U/ml)	259.0±609.5	122.5±289.0	<0.001
Mean (SD) BMI (kg/m^2^)	25.66±4.20	23.76±3.51	<0.001
Asthma symptoms			
Mean (SD) age at first diagnosis±SD	44.92±18.22	-	
In the past 12 months:			
Nocturnal waking with wheezing, %	231(38.5%)	-	
>1 attack/per week, %	287 (47.8%)	-	
Admission or emergency, %	78 (13.0%)	-	
Mean (SD) periods between the first time chewing and asthma diagnosed (yrs)	18.62±14.49	-	
Alcohol consumption (%)			
No	500 (83.5%)	1002 (84.4%)	0.656
Yes	99 (16.6%)	185 (15.6%)	
Smoking (%)			
No	454 (75.9%)	932 (77.8%)	0.001
Yes	144 (24.1%)	189 (15.8%)	
Betel chewing (%)			
No	532 (88.7%)	1115 (92.9%)	0.003
Yes	68 (11.3%)	85 (7.1%)	

Total IgE levels were logarithmically transformed before statistical testing to meet the assumption of a normal distribution.

**Table 2:** The associations of the duration, amount and total life time consumption of betel chewing and asthma.

Betel chewing		Total			Males			Females		
		Cases	Controls	p	Cases	Controls	p	Cases	Controls	p
Habit (%)										
	No	532(88.7%)	1115(92.9%)	0.001	192(75.3%)	426(83.5%)	0.02	340(98.6%)	689(99.9%)	0.031
	Yes	68(11.3%)	85(7.1%)		63(24.7%)	84(9.6%)		5(1.5%)	1(0.1%)	
Duration (%)										
	No	532(88.7%)	1115(92.9%)	0.01	192(75.3%)	426(83.5%)	0.02	340(98.6%)	689(99.9%)	0.02
	1–15 years	36(6.0%)	45(3.8%)		33(11.8%)	45(8.8%)		3(0.9%)	0(0.0%)	
	>15 years	32(5.3%)	40(3.3%)		30(12.9%)	39(7.6%)		2(0.6%)	1(0.1%)	
Mean (SD) age of first chewing		21.7±7.8	23.4±7.9	0.23	21.7±7.9	22.0±6.2	0.83	-	-	
Mean (SD) lifetime, pack×years		42.0±60.1	35.9±34.2	0.70	49.2±63.6	35.9±34.2	0.42	-	-	

Total life time exposure of betel chewing:one chewed pack corresponds to 10 betel quids.

In unadjusted analyses, therefore, BMI, smoking, betel chewing and eotaxin-1 levels were associated with asthma. In logistic regression analyses adjusting for the above potential confounding factors (Table 3), the former and current use of betel chewing were respectively associated with a 1.60- and 2.05-fold higher risk of asthma than having never used it. The attributable risk of asthma accounted for by betel chewing in the current or former use group was estimated as 0.435 (0.77/1.77). The percentage of the population attributable fraction was 4.9% ([68/600]×0.45) for all adult asthma, 11.7% for male asthma and 1.2% for female asthma. If eotaxin-1 was further included in the multiple logistic regression analysis, the association between current betel chewing and asthma was attenuated and not significant. Testing whether eotaxin-1 mediated the association between betel chewing to asthma, we found a Sobel-Goodman coefficient of 0.08 (p<0.001). Both a model with asthma regressed on betel chewing (p<0.001) and a model with log eotaxin-1 (mediator) regressed on betel chewing (p<0.001) were statistically significant. Furthermore, multiple regression with asthma regressed on log eotaxin-1 (mediator) and betel chewing was only significant for log eotaxin-1 (p<0.001), but the significance for betel chewing weakened (p = 0.045). The mediation effect of eotaxin-1 was significant, with approximately 50.5% of the total effect of betel chewing on asthma mediated through this pathway.

**Table 3:** Logistic regression analyses of the adjusted associations between betel chewing and asthma.

	All subjects (N = 1800)		Two-stage sampling (N = 734)					
Betel chewing	Crude Odds ratios (95% CI)	Adjusted Odds ratio 1 (95% CI)	Eotaxin-1 levels		Adjusted Odds ratio 1 (95% CI)	P-value	Adjusted Odds ratio 2 (95% CI)	P-value
			Asthma mean±SD	Control mean±SD				
			n = 345	n = 389				
No	1	1	175.6±71.8	131.5±53.1	1		1	
Former	1.75(1.14–2.69)	1.60(0.95–2.69)	232.5±126.5	179.6±42.4	2.47(1.04–5.84)	0.040	1.90(0.77–4.70)	0.166
Current	1.57(0.94–2.62)	2.05(1.12–3.76)	223.1±58.5	184.7±55.8	2.58(1.04–6.36)	0.038	1.64(0.62–4.37)	0.323
No	1	1	175.6±71.8	131.5±53.1	1		1	
Yes	1.68(1.20–2.34)	1.77(1.16–2.72)	228.1±99.6	181.8±47.3	2.52(1.27–4.99)	0.008	1.78(0.85–3.70)	0.12
Mediation	50.5% 3	p<0.001						

1 Logistic regression adjusted for age, gender, BMI and smoking.

2 Logistic regression adjusted for age, gender, BMI, smoking and log eotaxin-1 levels (345 cases and 389 controls were randomly selected to measure eotaxin-1 levels).

3 The mediation effect of eotaxin-1 was 50.5% of the total effect of betel chewing on asthma being mediated through this pathway.

In Table 4, four groups of male participants are compared according to the presence of asthma and current betel chewing after adjusting for potential confounding factors. Within the case group, arecoline, arecaidine, eotaxin-1 levels, FEV1 and FVC were significantly higher in people reporting current betel chewing. However, no associations were found between betel chewing and levels of leptin, transforming growth factor β1 (TGF-β1), or hs-CRP in asthmatics. In Table 5, a higher arecoline level was associated with higher eotaxin-1 levels and also associated with worse respiratory function in asthmatics. Higher arecaidine levels were only associated with a worse respiratory function in the case group.

**Table 4:** Associations between asthma, betel chewing, plasma markers and respiratory function in male participants (n = 147).

	Mean±SD levels		P a	Mean±SD levels		P a	ANOVA P-value	General linear model b P-value
	Male asthma			Male control				
	Current betel chewing (n = 21)	Non-current betel chewing (n = 42)		Current betel chewing (n = 32)	Non-current betel chewing (n = 52)			
Arecoline (ng/ml)	8.67±17.1	0.03±0.12	<0.001	14.1±39.6	0.26±0.66	<0.001	<0.001	<0.001
Arecaidine (ng/ml)	40.0±98.6	0.21±0.36	<0.001	18.0±63.5	0.63±2.11	0.01	<0.001	0.001
Eotaxin-1 (pg/ml)	229.1±57.4	184.9±60.2	0.03	179.3±51.2	164.1±80.4	0.15	0.002	0.002
FEV1, % predicted	77.6±18.0	92.4±17.9	0.002	87.0±17.6	93.3±14.9	0.15	0.008	0.014
FVC, % predicted	80.7±18.2	95.8±22.7	0.003	97.9±17.9	96.6±15.0	0.77	0.007	0.003
IgE (U/ml)	114.6±120.1	236.6±309.2	0.16	109.3±148.0	210.9±422.3	0.23	0.09	0.026
hs-CRP(mg/L)	1.60±1.76	2.27±3.84	0.97	0.94±0.92	1.21±1.22	0.34	0.32	0.336
Leptin (μg/ml)	6.23±3.97	5.07±3.69	015	-	-	-	-	-
TGF-β1 (μg/ml)	9.30±4.75	10.48±6.35	0.61	-	-	-	-	-

Arecoline, arecaidine, IgE, hs-CRP, leptin, TGF-β1 and eotaxin-1 levels were logarithmically transformed before statistical testing to meet the assumption of a normal distribution.

a Least significant difference (LSD) multiple comparisons were performed to compare plasma markers and pulmonary function between current and non-current betel chewing in males with asthma and controls, respectively.

b General linear regression was used to assess the associations between plasma markers and the four groups of male cases and controls with and without betel chewing by adjusting for age, BMI and smoking.

**Table 5:** Correlation of arecoline and arecaidine levels with eotaxin-1 levels and lung function in male asthmatics.

	Male asthma			
	Arecoline		Arecaidine	
	r*	p-value	r*	p-value
Eotaxin-1	0.303	0.02*	0.162	0.23
FEV1, % pred	−0.359	0.004*	−0.370	0.003*
FVC, % pred	−0.309	0.02*	−0.303	0.02*
IgE	0.103	0.43	−0.129	0.32
hs-CRP	0.042	0.78	0.060	0.68
Leptin	0.012	0.93	0.218	0.09
TGF-β1	−0.084	0.52	−0.145	0.26

*Spearman correlation coefficient.

Arecoline, arecaidine, IgE, hs-CRP, leptin, TGF-β1 and eotaxin-1 levels were logarithmically transformed before statistical testing to meet the assumption of normal distribution.

**Figure 1:**
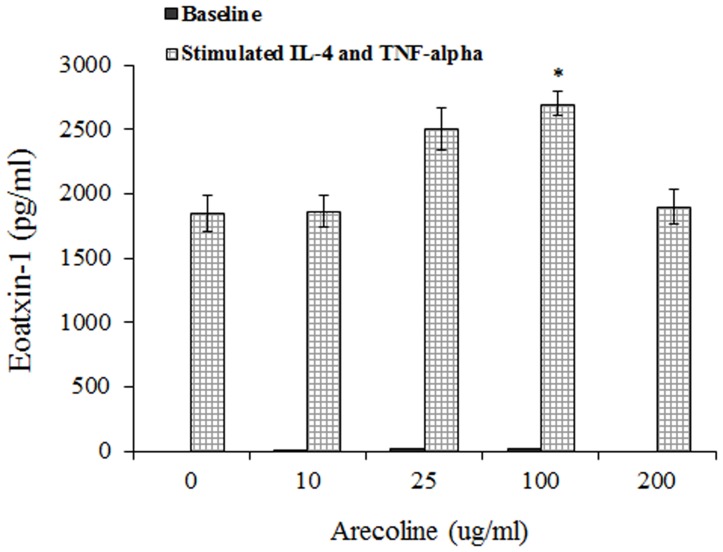
In cultured dermal fibroblast cells, the levels of eotaxin-1 were measured by ELISA in supernatants from cells treated with different levels of arecoline (0, 10, 25, 100, and 200 μg/ml for 24 h). The levels of eotaxin-1 (mean±SD) were analyzed by one-way ANOVA and Bonferroni multiple correction tests in which the P values are adjusted by multiplying by 10. * p = 0.01035 for the pretreatment of arecoline at 100 μg/ml induced significant elevation of eotaxin-1 levels (2700±98 pg/ml) compared with the pretreatment of arecoline at 0 μg/ml (1850±142 pg/ml) under the TNF-alpha and IL-4 stimulations. Arecoline increased cytotoxicity with more than 45% at a concentration of 200 μg/ml [22]. The stimulated groups indicate that the fibroblast cells were treated with IL-4 (50 ng/ml) and TNF-alpha (100 ng/ml) for 72 hours. The baseline groups are fibroblast cells that were treated with arecoline alone, not stimulated with IL-4 and TNF-alpha.

**Figure 2:**
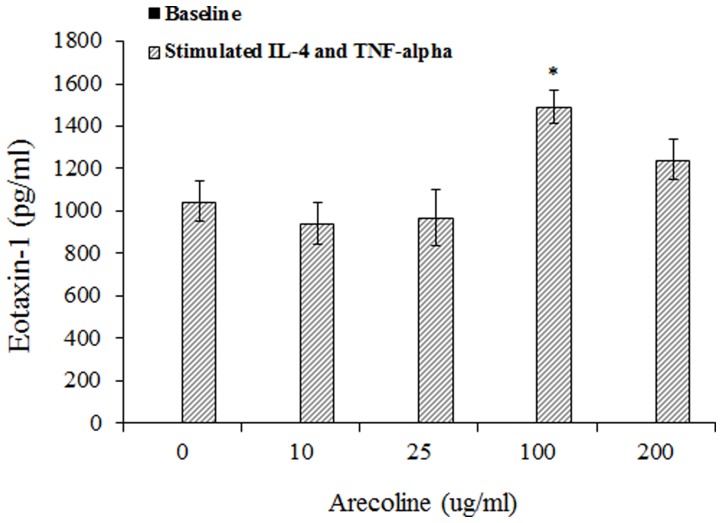
In cultured gingival fibroblast cells, the levels of eotaxin-1 were measured by ELISA in supernatants from cells treated with different levels of arecoline (0, 10, 25, 100, and 200 μg/ml for 24 h). The levels of eotaxin-1 (mean±SD) were analyzed by one-way ANOVA and Bonferroni multiple correction tests in which the P values are adjusted by multiplying by 10. * p = 0.03301 for the pretreatment of arecoline at 100 μg/ml induced significant elevation of eotaxin-1 levels (1489±78 pg/ml) compared with the pretreatment of arecoline at 0 μg/ml (1044±95 pg/ml) under TNF-alpha and IL-4 stimulation. Arecoline, at a concentration of 200 μg/ml, increased cytotoxicity by more than 45% [22]. The stimulated groups indicate that the fibroblast cells were treated with IL-4 (50 ng/ml) and TNF-alpha (100 ng/ml) for 72 hours. The baseline groups are fibroblast cells that were treated with arecoline alone, not stimulated with IL-4 and TNF-alpha. Arecoline alone at any tested concentrations from 0 to 200 μg/ml cannot induce detectable eotaxin-1 release (0±0 pg/ml for any tested dose of arecoline).

To investigate the functional associations between arecoline exposure and eotaxin-1 levels, we harvested fibroblasts with or without stimulations with IL-4 and TNF-alpha for 72 hours and measured eotaxin-1 in the conditioned medium. We found that treatment with arecoline alone at tested concentrations of 0, 10, 25, 100 and 200 μg/ml induced very little eotaxin-1 release (from 0±0 pg/ml to 23±2 pg/ml of eotaxin-1 for any tested dose of arecoline). However, we found that pretreatment with arecoline at 100 μg/ml induced a significant elevation of eotaxin-1 levels (2700±98 pg/ml, p = 0.01 by Bonferroni multiple correction tests) compared with the pretreatment of arecoline at 0 μg/ml (1850±142 pg/ml) under the TNF-alpha and IL-4 stimulations. A drop in eotaxin-1 level (1898±132 pg/ml) was noticed in stimulated fibroblasts treated with 200 μg/ml arecoline (Figure 1). Because the exposure to betel chewing directly interacts with the oral mucosa but not the skin, we harvested gingival fibroblasts and repeated the above experiment to consolidate the cause-relationship. We also found that treatment with arecoline alone at any tested concentration from 0 to 200 μg/ml did not induce an eotaxin-1 release (0±0 pg/ml for any tested dose of arecoline). In the presence of TNF-alpha and IL-4, the pretreatment of arecoline at 100 μg/ml induced a significant elevation of eotaxin-1 expression (1489±78 pg/ml) compared with the retreatment of arecoline at 0 μg/ml (1044±95 pg/ml, p = 0.03) from stimulated gingival fibroblasts (Figure 2).

## Discussion

To date, few epidemiological studies have investigated the potential effect of betel chewing on the susceptibility to asthma and impairment of lung function, and these studies have suffered from relatively small sample sizes [10], [19]. The underlying mechanism that drives the proposed relationship between betel chewing and asthma is also still unclear. A causal role of betel chewing on asthma risk is supported by a strong relationship of this exposure as well as exposure to arecoline and eotaxin-1 with asthma, both in our large case-control study and in a supplementary *in vitro* experiment. There are several novel findings in the present study. The first is that the arecoline levels were positively correlated with eotaxin-1 concentrations and negatively correlated with lung function in male cases. Second, current betel chewing was associated with asthma, but this association was attenuated after adjustment for eotaxin-1 concentrations. Third, the functional connection between arecoline exposure and eotaxin-1 release was supported by the *in vitro* experiment. Therefore, our findings support an effect of betel chewing on asthma that is mediated at least in part by eotaxin-1.

In a previous double blind challenge study, the concentration of arecoline that caused a 20% reduction in FEV1 (PC20) in asthmatic patients was 5.2 mg/ml, and the PC20 value was only 1.6 mg/ml for methacholine, which exerted a stronger effect than arecoline [10]. A skin prick test in patients with a respiratory allergy showed a 38.6% positivity against areca pollen, and this was significantly associated with emergency asthma hospitalization [19]. Tracheal contraction to both histamine and arecoline increased significantly in ovalbumin-aerosol exposed animals [20], and was enhanced by 155% compared to saline-aerosol exposed animals [21]. In our study, current betel chewing increased the risk of asthma, and the levels of arecoline and arecaidine were negatively correlated with FEV1 and FVC measures of respiratory function in male asthmatic patients. Therefore, the present study is consistent with previous findings.

To our knowledge, ours is the first study to investigate the relationship between the levels of arecoline, asthma and eotaxin-1. We demonstrated that the levels of arecoline were positively correlated with eotaxin-1 *in vivo* and that eotaxin-1 release could be stimulated significantly by pretreatment with arecoline at 100 μg/ml in the presence of IL-4 and TNF-alpha. A notable pathological atrophy of cell morphology was found with arecoline concentrations of 100 μg/ml, which is consistent with a previous study that also suggested that the effective concentration of arecoline was approximately 100 μg/ml [22]. Eotaxin-1 is a chemokine that can regulate nuclear factor kappa B (NF-κB) and is stimulated by cytokines and oxidant stress [23], [24]. Hydroxyl radicals, superoxide radicals and H_2_O_2_ are found in the gas phase of environmental tobacco smoke (ETS) [25] and are also formed while chewing betel quid [26]. Reactive oxygen species (ROS) are known to activate regulators of eotaxin-1 expression, such as NF-κB [27]. NF-κB activation and ROS genesis have both been found to be induced by areca nut extract; therefore, the NF-κB activation may be the basis of the ROS genesis [28]. Several animal studies have reported that smoke and environmental tobacco smoke increase the levels of eotaxin-1 expression and allergen-induced airway remodeling [29], [13]. Therefore, we suggest that the mechanisms of exposure to ETS and arecoline may implicate similar mechanisms of asthma pathogenesis.

Our *in vitro* study found that treatment with arecoline alone by dermal and gingival fibroblasts at any concentration did not induce eotaxin-1 release. However, after stimulation with IL-4 and TNF-alpha, both of which are standard agents to induce robust release of eotaxin-1 from fibroblasts [17], [18], pretreatment with arecoline at 25 and 100 μg/ml induced a significant elevation of eotaxin-1, suggesting that under proinflammatory circumstances, arecoline can induce eotaxin-1 release and modify the disease process in asthma. Similar to our study, mice exposed to chronic ETS alone did not develop significant airway inflammation, but co-exposure to a combination of ETS and OVA allergen induced increased expression of eotaxin-1 compared with exposure to either chronic ETS or chronic OVA allergen alone [29]. In our study, a drop in the eotaxin-1 level was noticed in stimulated fibroblasts treated with 200 μg/ml arecoline compared with the pretreatment of arecoline at 100 μg/ml (Figure 1 and 2), which may be caused by the toxicity of arecoline to fibroblasts in vitro. Our previous cytotoxic assay of arecoline found a more than 45% increase of cytotoxicity with arecoline treatments at a concentration of 200 μg/ml [22].

### Strength and limitations of study

There are several noteworthy strengths in the present study. First, we applied an epidemiological study to test the primary hypothesized relationship between betel chewing and asthma as well as to investigate whether eotaxin-1 levels explain the association of betel chewing with asthma, which was supplemented by an *in vitro* experiment to demonstrate the functional link between arecoline pretreatment and a significant elevation of eotaxin-1 using cultured cell lines of both human normal dermal and gingival fibroblasts. Dermal fibroblasts are a potent source of human eotaxin-1 [30]. Second, a temporal relationship between betel chewing as an exposure and asthma as an outcome was supported because the mean±SD age of first reported betel chewing in asthma patients was 21.7±7.8 years compared to a mean±SD age of first asthma diagnosis of 44.9±18.2 years. Only three cases reported the latter to be earlier than the former, and most reported a long period of prior betel exposure. Third, logistic regression models were used to control for potential confounding factors, including age, BMI, smoking and betel chewing, which were associated with the case status, consistent with reports from previous studies. Fourth, although the measurement of betel chewing was based on self-reported information with the attendant risk of recall bias, arecoline and arecaidine were also significantly higher in people reporting current betel chewing in asthma and the control groups in this study. We have previously found that plasma arecoline and arecaidine levels are measurable and good biomarkers for recent betel use [8], thereby improving the exposure ascertainment in this respect.

Several limitations of this study should be considered. The first limitation is a very low prevalence of betel chewing in Taiwanese females, as previously reported [31], leading to a difficultly in investigating the association with asthma in females, although case control differences were confirmed in women in unadjusted analyses. Second, it was difficult to obtain direct information on past arecoline exposure from a case control study; hence, we only evaluated the arecoline exposure for current chewers and not for former chewers. However, we analyzed the associations between former betel chewing and asthma, and these were consistent with, although weaker than, those with current chewing (Table 3). In sum, we do not believe that inaccurate or biased recall accounted for the observed associations. In this study, our results suggest a population attributable fraction for betel chewing on asthma of 11.7% for males and 1.2% for females, and our findings strongly implicate a relationship between arecoline levels and eotaxin-1 concentrations, providing further evidence for a major role for eotaxin-1 in linking betel chewing with asthma.

## Supporting Information

File S1 Methods supplement.
(DOC)
