# Supplementary material for: Betel Chewing and Arecoline Affects Eotaxin-1, Asthma and Lung Function
Source: PLoS One. 2014 Mar 21;9(3):e91889. doi: 10.1371/journal.pone.0091889 (PMC3962362; doi:10.1371/journal.pone.0091889)
Supplement: File S1 — Methods supplement. (DOC) [file pone.0091889.s001.doc]

**Online data supplement**

[**Betel chewing and arecoline affects eotaxin-1, asthma and lung function**](http://www.ncbi.nlm.nih.gov/pubmed/21565829)

Tsu-Nai Wang, PhD, Ming-Shyan Huang, MD, PhD, Meng-Chih Lin, MD, Tsai-Hui Duh, PHD, Chih-Hung Lee, MD, PHD, Chin-Chou Wang, MD, PHD, Ping-Ho Chen, PHD, Shang-Lun Chiang PHD, Chau-Chyun Sheu, MD, Vincent Chin-Hung Chen, MD, PHD, Chao-Chien Wu, MD, Cleusa P. Ferri, MD, PHD, Robert Stewart, MD, Ying-Chin Ko, MD, PHD

**METHODS – Supplement**

**The demographic information and lung function measurements**

All participants completed a questionnaire which included demographic characteristics, occupational history, indoor environmental allergies to pets, mold, cockroaches and medical history. Occupational history included previous and current job history, job title and working years. Forced vital capacity (FVC) and forced expiratory volume in the first second (FEV1) were measured to assess pulmonary function. Pulmonary function tests were performed according to the recommendations of American Thoracic Society [1], and the highest score for each of the three tests was recorded. Blood samples were collected in heparin-containing venipuncture tubes.

**Measurement of smoking, alcohol and betel chewing**

Information was collected on smoking, alcohol use and betel chewing, including age at first use, frequency of consumption and duration of use. Definitions of cigarette smoking, alcohol consumption and betel chewing were applied as previously published [2]: i) consumption of any alcoholic beverages ≥1 time per week for a minimum of 6 months, ii) smoking of ≥10 cigarettes per week for a minimum of 6 months, and iii) chewing of ≥1 betel nut quid per day at least 6 months. Former users of each were those who had stopped cigarette smoking, alcohol consumption and betel chewing for at least 6 months prior to the interview. Those who chewed betel less than once per day or for less than six months were categorized as never users. To examine the effect of total lifetime exposure to betel chewing, an equivalent of ‘‘pack × years’’ was calculated by multiplying the amount of the betel nuts consumed by the duration of consumption, with one ‘pack’ defined as 10 betel quids,.

**High sensitive CRP (hs-CRP) and eotaxin-1 (CCL11) levels**

Two-stage random sampling was performed to select 350 cases and 400 controls among all study participants to measure eotaxin-1 and hs-CRP levels. Of these, 5 cases and 11 controls had insufficient plasma for eotaxin-1 and hs-CRP levels detection. Therefore, 345 cases and 389 controls were compared using logistic regression models before and after controlling log-eotaixin. Human plasma was stored at -70^o^C before assay of hs-CRP and eotaxin-1 levels. Human plasma hs-CRP concentrations were measured using a solid-phase, chemiluminescent immunometric assay (IMMULITE/IMMULITE 1000 Analyzer, Diagnostic Products Corporation, Los Angeles, CA, USA) with 0.2 mg/L of the lowest detectable level of hs-CRP. Human plasma eotaxin-1 levels and eotaxin-1 release of stimulated fibroblasts in the conditioned media were measured by ELISA from a commercially available ELISA kit (R&D System, Minneapolis, MN, USA) based on the manufacturer’s instructions. Total plasma IgE was measured by using a solidphase, chemiluminescent immunometric assay (IMMULITE 2000 Analyzer, Diagnostic Products Corporation, Los Angeles, CA, USA.).

References

1. American, Thoracic, Society (1987) Standardization of spirometry--1987 update. Statement of the American Thoracic Society. Am Rev Respir Dis 136:1285-1298

2. Lee CH, Ko YC, Huang HL, Chao YY, Tsai CC, et al. (2003) The precancer risk of betel quid chewing, tobacco use and alcohol consumption in oral leukoplakia and oral submucous fibrosis in southern Taiwan. Br J Cancer 88:366-372
